# Unusual Cutaneous Location of Langheransian Histiocytosis: A Case Report

**DOI:** 10.7759/cureus.19617

**Published:** 2021-11-16

**Authors:** Mounia Bendari, Idriss Chamizakhraji, Saloua Elamari, Kenza Oqbani, Maryame Ahnach

**Affiliations:** 1 Hematology Department, Cheikh Khalifa International University Hospital, Mohammed VI University of Health Sciences (UM6SS), Casablanca, MAR; 2 Hematology Department, Faculty of Medicine, Mohammed VI University of Health Sciences (UM6SS), Casablanca, MAR; 3 Endocrinology Department, Faculty of Medicine, Mohammed VI University of Health Sciences (UM6SS), Casablanca, MAR; 4 Pathology Department, Faculty of Medicine, Mohammed VI University of Health Sciences (UM6SS), Casablanca, MAR

**Keywords:** relapse, chemotherapy, skin involvement, langerhans cells histiocytosis, adult

## Abstract

Langheransian cell histiocytosis (LCH) is a rare pathology characterized by the proliferation of CD1+ and Langerin+ cells. It can affect all ages, with an estimated prevalence of one to two cases/100,000 habitants. The involvement is often multi-visceral; however, isolated cutaneous involvement can be found in 40% of cases with very variable manifestations. We report the case of 45-year-old women followed for non-insulin-dependent diabetes and primary hyperparathyroidism suffering from isolated and refractory cutaneous histiocytosis.

## Introduction

Paul Langerhans was the first to describe the Langerhans cell in 1868 [1]. Alfred Hand reported the first definitive case of Langerhans cell histiocytosis (LCH) in 1893 [2]. It mainly affects children under three years of age (4 to 5 per million children 0 to 15 years of age each year) and the incidence in adults is around 1-2/million; it appears that the incidence is higher in Caucasians in northern European countries and lower in Asia and Africa [3-4]. LCH is characterized by variable clinical presentation and heterogeneous prognosis with common histopathological aspects; it’s characterized by an infiltration of the body's tissues by atypical Langerhans cell histiocytes, often organized in granulomas. Langerhans cells or histiocytes are immature dendritic cells that are found mainly in the skin and the multilayered mucous membranes of the body [5-6]. Clinical presentations are very diverse, ranging from a single lesion to severe multi-visceral forms. We report the case of 45-year-old women followed for non-insulin-dependent diabetes and primary hyperparathyroidism with refractory and multifocal cutaneous histiocytosis. Through our case, we illustrate the particularity of cutaneous presentations of histiocytosis and underline the real challenge in the diagnosis and management of this rare entity. The major aim of our report is to describe the characteristics of the cutaneous form of this disease, especially when they are refractory.

## Case presentation

**Figure 1:**
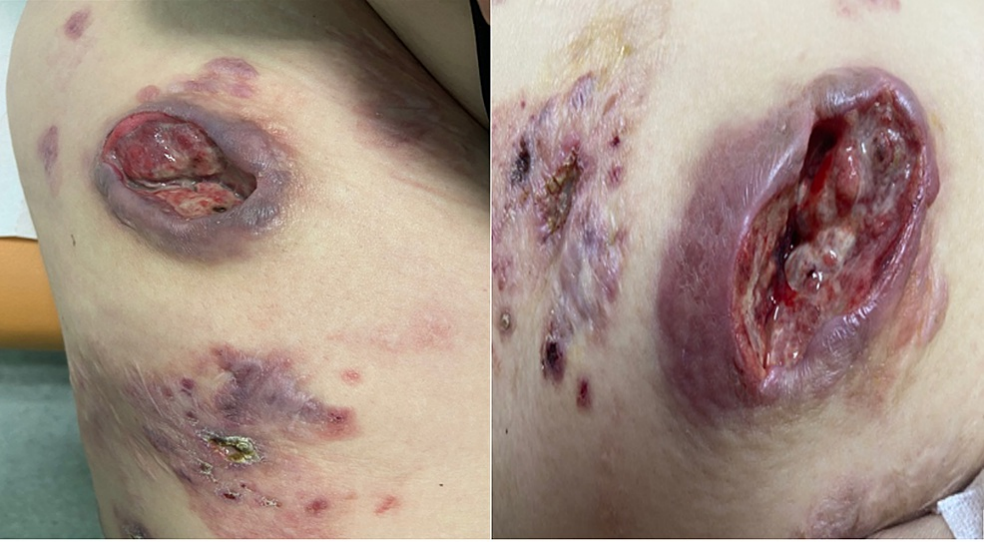
Skin lesion of LCH Ulcers and necrotic lesions, purplish, well-limited, of variable size, not painful, not warm to the skin, and localized to the right thigh LCH: Langheransian cell histiocytosis

We report a case of a 45-year-old woman, a non-smoker, treated for type II diabetes under insulin and primary hyperparathyroidism. Her medical history dates back to 2006 with an infiltrated nodule associated with ulcers that grew gradually in her right thigh. The patient underwent surgery with an anatomopathological study. Skin biopsy objectified ulcerated epidermotropic dermo-hypodermal tumor proliferation whose morphological appearance and immunohistochemical data are in favor of LCH. Langerhans cells present positivity of the anti-PS100 antibody, anti-CD1a antibody, and anti-Ki67 antibody (60%), and negativity of the anti-CD68 antibody. The patient subsequently received 25 sessions of radiotherapy followed by six courses of chemotherapy with a low dose of oral methotrexate. After these treatments, skin lesions are stable. In 2020, the patient complained of an increase in skin lesions, with the appearance of several infiltrating nodules, scaling, crusted papules, and ulcerated plaques. The patient received chemotherapy as single-system LCH (SS-LCH) based on methotrexate, associated with prednisone and vinblastine, with the obtainment of stable response and limited regression of the lesions. After two courses, the patient was lost to follow-up. One year later, the oncologist referred the patient to the hematology department for a major increase in skin lesions, with the appearance of new ones on her thigh. Dermatological examination found ulcers and necrotic lesions, purplish, well-limited, of variable size, not painful, not warm to the skin, and localized to the right thigh. The mucous membranes and integuments were unharmed (Figure 1).

**Figure 2:**
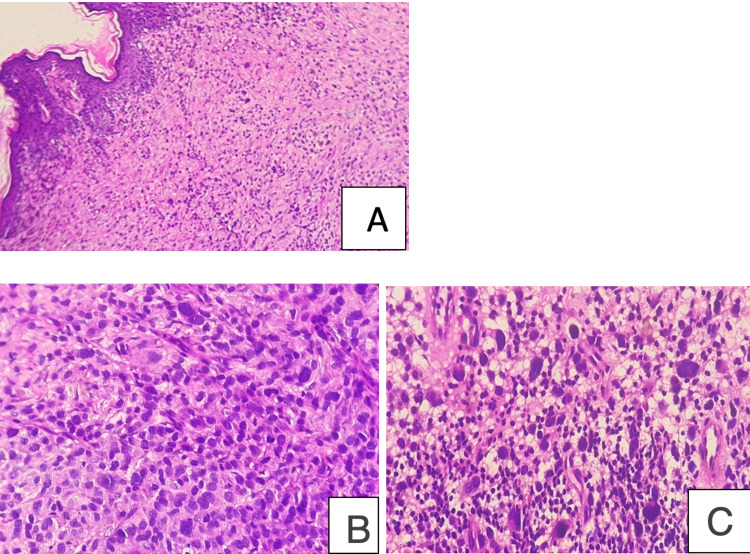
Langerhans cell (LC) sarcoma, microscopic exam A: large cells dermal infiltrate (low power field H&E); B: irregular and elongated nuclei with prominent nuclear grooves and folds (high power H&E); C: nuclear pleomorphism and variable inflammatory infiltrate surrounding neoplastic tumor cells (high power H&E)

Furthermore, the examination found an afebrile, obese patient (body mass index (BMI) at 44.8). Abdominal and cardiovascular examinations were unremarkable. The physical examination did not find any tumoral syndromes, and the patient did not present have B signs (fever, weight loss, and night sweats). A complete assessment was carried out in favor of a cutaneous location without visceral involvement. A CT scan of the chest and abdomen was normal. Extensive investigation revealed no systemic involvement, central nervous system (CNS) evaluation was not done, serum thyroid hormone levels were normal, endocrine workup was performed, including cortisolemia, and parathormone assessment were done in favor of primary hyperparathyroidism. A skin biopsy was performed. The microscopic examination revealed a cutaneous tissue with an infiltrate of large cells involving the dermis and realizing an epidermotropism. The infiltrate was composed of atypical cells with abundant, pale eosinophilic cytoplasm, irregular and elongated or lobed nuclei with prominent nuclear grooves and folds, fine chromatin, and indistinct nucleoli. Multinucleated giant tumor cells, high cytonuclear atypia, and marked nuclear pleomorphism were seen. The mitotic figures were increased. The neoplastic cells also showed a mixed inflammatory background with variable numbers of reactive lymphocytes, plasma cells, benign histiocytes, and eosinophils associated (Figure 2).

**Figure 3:**
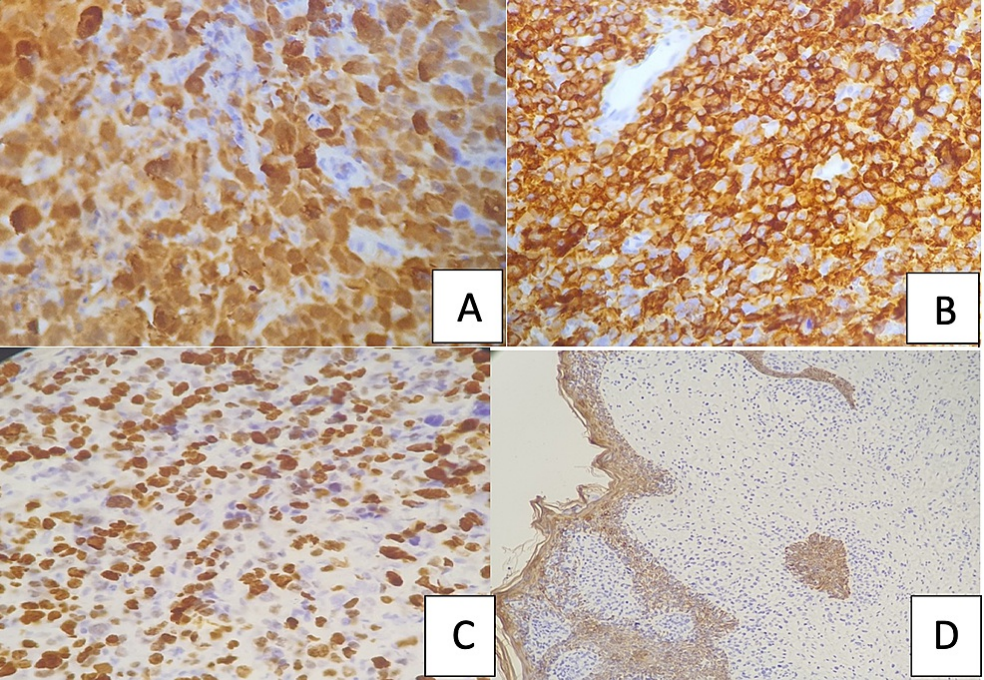
Langerhans cell (LC) sarcoma, immunohistochemistry A: S100 was widely represented, nuclear and cytoplasmic, but variable in intensity. B: Neoplastic cells expressed surface CD1a. C: Ki-67 stain showed a high proliferation rate. D: Tumor cells were negative for cytokeratins (Note the epithelial cells positive control).

The immunohistochemical stains had shown a diffuse strong and heterogeneous positivity for CD1a and S100 and variable positivity for CD45. All other markers studied were negative, including CD68, cytokeratins (AE1/AE3), and melanocytic marker (MelanA). The Ki-67 proliferation index was high (50%) (Figure 3).

**Figure 4:**
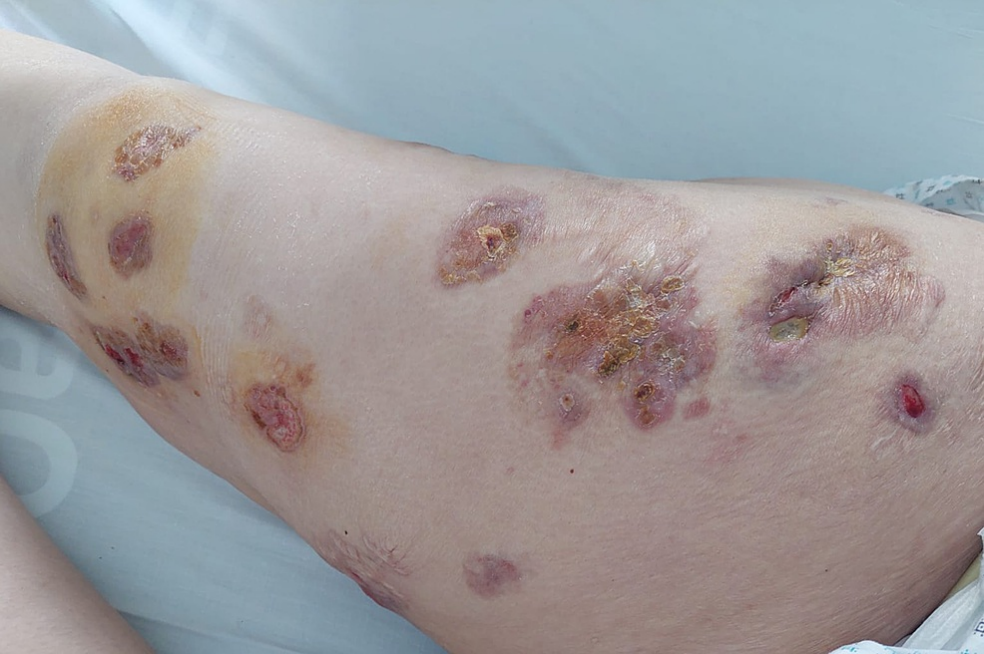
Skin lesions after three courses of chemotherapy Favorable response after chemotherapy

In the light of microscopic and phenotypic results, the diagnosis of Langerhans cell sarcoma was confirmed. The patient did not receive any maintenance therapy and she received only two courses of chemotherapy; consequently, the patient was included in the LCH protocol as single-system LCH (SS-LCH), and she received the same chemotherapy combining prednisone (60 mg / m^2^), high-dose of methotrexate (5000 mg/m^2^), and vinblastine (10 mg/m^2^). The evolution was favorable after five cycles of chemotherapy with significant regression of lesions in the thigh without the appearance of any new lesions (Figure 4).

## Discussion

Langheransian cell histiocytosis (LCH) is a pathology characterized by the proliferation of Langerhans cells in various organs, such as bone, liver, spleen, or even the skin. Involvement of the skin is found in 40% of cases; it mainly affects children [5,7]. In the 2016 revised classification of the Histiocyte Society, LCH is currently defined as an inflammatory myeloid tumor [8]. Among the most affected organs, the bone is the most touched (80%), the skin is also affected (40%), and the pituitary (25%), liver, spleen, lungs, and central nervous system can also be touched [9]. In general, the involvement is multisystem or multifocal, rarely isolated to a single organ. Bone and lungs are the two organs most affected in the localized form, unlike cutaneous histiocytosis, which is rarely isolated in adults and is mainly present in multisystem forms of the disease. In fact, the involvement is multisystem or multifocal, rarely isolated to a single organ. As mentioned in the observation of this patient, we report the case of isolated skin involvement; skin involvement manifested as ulcerative necrotic lesions of varying sizes. Likewise, during histiocytosis, it is usual to find diabetes insipidus due to pituitary involvement [1]. For our patient, she had type II diabetes with primary hyperparathyroidism. The cutaneous presentations can be very variable, lesions can be papular or papulovesicular, nodular, hemorrhagic, seborrheic, scaly, or ulcerated lesions. The scalp, folds of the body, external genitals, and hairless skin are the main sites of cutaneous damage [6]. Our patient had heterogenous skin lesions but all localized to her thigh. The diagnosis of histiocytosis is based on the histological examination of the affected organ, which shows a granuloma with Langerhans cells, the appearance of which varies depending on the stage of development and the tissue involved. The Langerhansian nature can be confirmed in immunohistochemistry by the expression of the membrane antigen CD1a or Langerin (CD207). The diagnosis of LCH is not easy; for our patient, many skin biopsies with immunohistochemistry studies were performed to confirm the diagnosis.

Significant advances have been made in understanding the pathogenesis of LCH since 2010 due to the discovery of the presence of the gain-of-function mutation in BRAF (V600E) in more than half of the samples from patients with LCH [10-11]. Since then, several studies have reported that 100% of LCH cases present with ERK phosphorus, suggesting that LCH is probably a clonally expanding myeloid neoplasm [12]. Moreover, the presence of the BRAFV600E mutation has been reported in tissue samples of half of the patients, and it has been shown that the activation of the RAS-RAF-MEK-ERK pathway is always present in LCH lesions, regardless of BRAF status. These results represent an important step in understanding the pathophysiology of the disease [13]. Regarding treatment, patients with single-systemic disease confined to a single site generally required only local treatment. Systemic treatment is possible in some cases for localized form [7]. In more extensive forms, systemic treatment is usually necessary [14-15]. In fact, the treatment of skin involvement is based on the excision of the lesion with topical corticosteroids if it is limited. For refractory forms, more complex protocols can be proposed (mono or poly-chemotherapy). The response of treatment is usually favorable for adults [6] and for isolated localization. However, our patient presented several relapses, leading to the use of several lines of therapy. Unfortunately, our patient was not tested for BRAF status. The treatment of LCH remains controversial. Indeed, therapeutic management depends on the number of sites affected, the severity of the lesions, and the age of the patient. Many therapies have been tried such as chemotherapy, radiotherapy, or surgery [16]. For our patient, after relapse, the LCH protocol (vinblastine and high-dose methotrexate (MTX)) was chosen, and permits having a favorable response. The aim of this report is to highlight the difficulties of diagnosis and management of this particular disease. Our patient is an adult woman, with only skin localization, which is rare for histiocytosis. Also, localization at the level of the thigh is not common for LCH.

## Conclusions

LCH is a rare pathology that has multiple clinical presentations. The diagnosis must be made with precision, and the extension assessment allows patients to be classified and treated according to whether the involvement is multifocal or limited to a single organ. The outcome is often favorable with treatment, but relapses can occur even in localized cases. Recent publications report the interest of the research on the BRAF (V600E) mutation to confirm the diagnosis of LCH.
